# NURSING HOME ADMINISTRATOR STRESS, SATISFACTION, AND INTENTIONS TO LEAVE

**DOI:** 10.1093/geroni/igz038.1864

**Published:** 2019-11-08

**Authors:** H Wayne Nelson, Bo Kyum Yang, Cyrus Y Engineer, Mary W Carter

**Affiliations:** Towson University, Towson, Maryland, United States

## Abstract

Previous studies reported that high Nursing Home Administrator (NHA) turnover correlates with low staff morale and poorer care outcomes. The purpose of this study was to assess how NHA job satisfaction (JS) (in five subscales: job content, coworkers, work demands, work load, work skills, and rewards) interacts with role conflict and ambiguity, autonomy, work conflict, and influence and to estimate the odds of having NHA’s intent to quit by degree of job satisfaction. A total 208 responses were collected from the online survey in 2017 among NHAs currently working in nursing homes in 5 states. We analyzed the data using descriptive statistics and ordinal logistic regression models. The findings suggested that NHAs were generally satisfied in all JS subscales and expressed moderately high levels of autonomy, neutral levels of work conflict, role conflict and role ambiguity. NHAs with good coworker relations (adjusted odds ratio [AOR]=0.67), fair job demands (AOR=0.68) and rewards (A0R=0.8), were less likely to harbor quitting intents. Interestingly, NHAs reporting higher job skills were more likely to consider leaving nursing homes (AOR=1.46). Overall, study findings are consistent with previous JS research with the exception that higher perceived skill efficacy was found to be associated with greater likelihood of quitting in the near future. This suggests perhaps that more highly skilled NHAs may now have less tolerance for work discomfort. These findings are presented in the context of earlier studies on NHA turnover as well as likely implications of changing market conditions.

